# A Diagram of the Social-Ecological Conditions of Opioid Misuse and Overdose

**DOI:** 10.3390/ijerph20206950

**Published:** 2023-10-20

**Authors:** Benjamin R. Brady, Ehmer A. Taj, Elena Cameron, Aaron M. Yoder, Jennifer S. De La Rosa

**Affiliations:** 1 Comprehensive Pain and Addiction Center, University of Arizona Health Sciences, Tucson, AZ 85721, USA; ehmert@arizona.edu (E.A.T.); ercameron@arizona.edu (E.C.); jschult1@arizona.edu (J.S.D.L.R.); 2 School of Interdisciplinary Health Programs, College of Health and Human Services, Western Michigan University, Kalamazoo, MI 49008, USA; 3 Comagine Health, Seattle, WA 98133, USA; aaronyoder@arizona.edu; 4 Lifecourse Epidemiology of Adiposity and Diabetes (LEAD) Center, University of Colorado, Aurora, CO 80045, USA; 5 Department of Family and Community Medicine, College of Medicine, University of Arizona, Tucson, AZ 85721, USA

**Keywords:** opioid use disorder, overdose, social determinants, social ecology

## Abstract

The United States is experiencing a crisis of opioid misuse and overdose. To understand the underlying factors, researchers have begun looking upstream to identify social and structural determinants. However, no study has yet aggregated these into a comprehensive ecology of opioid overdose. We scoped 68 literature sources and compiled a master list of opioid misuse and overdose conditions. We grouped the conditions and used the Social Ecological Model to organize them into a diagram. We reviewed the diagram with nine subject matter experts (SMEs) who provided feedback on its content, design, and usefulness. From a literature search and SME interviews, we identified 80 unique conditions of opioid overdose and grouped them into 16 categories. In the final diagram, we incorporated 40 SME-recommended changes. In commenting on the diagram’s usefulness, SMEs explained that the diagram could improve intervention planning by demonstrating the complexity of opioid overdose and highlighting structural factors. However, care is required to strike a balance between comprehensiveness and legibility. Multiple design formats may be useful, depending on the communication purpose and audience. This ecological diagram offers a visual perspective of the conditions of opioid overdose.

## 1. Introduction

Opioids have a lengthy history of healing and harm. In the past century, shifts in political, social, and medical perspectives on opioids have led to changes in availability, meaning, and demographic use patterns [1]. From morphine to Oxycontin, clinicians and consumers have been awed by opioid’s analgesic capacity and tempted by their euphoric reward. This trend has continued in the 21st century. The United States’ current opioid crisis began with prescription overprescribing in the 1990s, and as opioid use and addiction escalated, prescription misuse spilled into illicit sources—heroin and synthetic derivatives [2]. The result has been a crisis of overdose and death.

Opioid misuse and overdose pose a major public health burden in the United States. Between 1991 and 2013, the prevalence of non-medical prescription opioid use increased from 1.5% to 4.1% [3,4]. From 2015 to 2021, as use shifted to fentanyl and other synthetics, deaths increased 7.5-fold [5]. While restrictions on opioid prescribing became more stringent, opioid [6] overdose deaths increased from around 21,000 in 2010 to over 90,000 in 2021 [5].

To address the opioid crisis, more attention has been given to social and structural determinants [7]. This focus facilitates a more comprehensive understanding of the multiple and intersecting risk factors that occur across social and environmental levels of influence. In recent years, the number of studies that identify social determinants of opioid use has increased. In PubMed, the search “opioid social determinant” yielded 58 articles in 2010 and 521 in 2022 [8]. In this literature, researchers highlight socioeconomic factors [7,9], community characteristics [10], social capital [11], and criminal justice involvement [12,13], to name a few.

To understand how the determinants of opioid use interact, it is helpful to synthesize and combine them into a comprehensive model. The social ecological model provides a useful framework to do this. The Centers for Disease Control and Prevention (CDC) highlights four levels of social ecology: individual, interpersonal, community, and societal [14]. Applied to the opioid crisis, this framework illustrates essential factors that explain opioid exposure, use, harm, and overdose [15].

Beyond locating social determinants into levels, an ecological diagram can also map the relationships among multiple determinants to clarify how they are interconnected and associated with health condition outcomes. In the tradition of theory-driven evaluations, conceptually mapping the array of antecedent conditions provides context and reveals factors that are potentially ignored when designing interventions and policy [16]. Social ecological diagrams thus emerge as powerful tools for program and policy design by clarifying and making explicit the assumptions that underly an intervention’s “theory of change” [17]. Visual diagrams are an effective way to aggregate information and model the ecosystem of factors that contributes to a problem and highlight logical connections between them. Visually modeling complex phenomena facilitates clearer communication and multimodal thinking [18].

Given the efficacy of diagrams and the need for a coherent synthesis of the factors surrounding opioid overdose, the aim of this project is to document and diagram the social ecology of opioid use, misuse, and overdose. We scoped the literature to document conditions identified in prior research. To explore their connections, we organized identified conditions into categories and used the levels of the Social Ecological Model to logically arrange them within a visual diagram. We reviewed the diagram with subject matter experts and updated it based on their feedback. Our aim is to provide a visual tool that can be used to inform and facilitate the development and design of more effective clinical practices, programs, and policies intended to improve the opioid crisis.

## 2. Materials and Methods

### 2.1. Literature Review and Diagram Creation

#### 2.1.1. Literature Search Parameters

We used a scoping review approach to identify the underlying conditions of opioid misuse and overdose. Scoping reviews are ideal for identifying and mapping available evidence [19,20]. This includes identifying key concepts, clarifying characteristics related to them, and finding and analyzing knowledge gaps [21]. Scoping reviews allow researchers to cover broad sets of literature and provide a macroscopic view of what is known. This fits the purpose of our project. In our review, we followed the steps outlined by Levac et al. [22] and Daudt et al. [23].

After defining our research question, we located resources from several locations: Google’s web browser, Google Scholar, two scholarly journal databases—PubMed and EMBASE—and through snowball and handsearching. We reviewed the first 50 hits from the two Google-based searches and included all the results from the journal database searches. We used Google searching to broaden and identify additional sources that are not commonly present in scholarly databases, like governmental literature [24]. In searching, we restricted to records published in English and in 2010 or later. In PubMed, we searched titles and abstracts, and in PubMed and EMBASE, we used the following key word combinations: ((opioid*, OR opioid misuse OR opioid overdose) AND (cause* OR factor* OR reason* OR determinant*).” In Google and Google Scholar, we searched using the terms “opioid use overdose cause* factor* reason* determinant*)”. We used asterisks (*)in these searches as a wildcard operator. It allows for a broader search by substituting for multiple characters like plurals, gerunds, or other endings.

#### 2.1.2. Literature Sample

To be eligible, a record could be a government report, industry/agency white paper, magazine article, scientific/peer reviewed study, scientific letter/opinion paper, government web page, or dissertation paper. We required that records utilize scholarly citations or be peer reviewed. However, consistent with scoping review methodology, we did not evaluate the quality of the studies. Instead, the emphasis was on identifying and classifying a range of information related to our topic [20].

**Figure 1:**
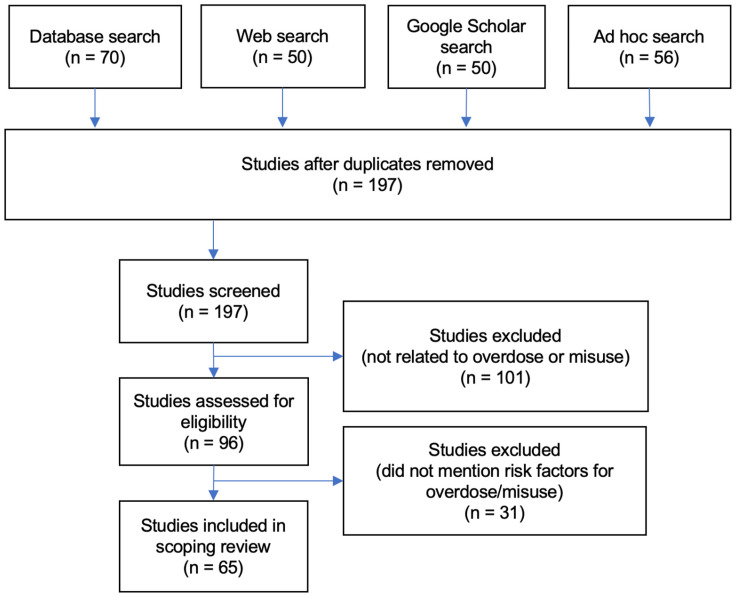
Literature selection and inclusion criteria.

Records were selected through an iterative review with three researchers. As shown in Figure 1, we removed 29 duplicate records and screened the remaining to assess eligibility. In screening, we examined titles and abstracts. Sources that did not relate to opioid use or overdose or that were related to medicinal opioid use were excluded. The remaining sources were further assessed for eligibility and excluded if they did not list causes, predictors, or risk factors for opioid use or overdose.

#### 2.1.3. Content Analysis

Data for this project included conditions that explain or predict opioid use and overdose. The names of conditions were extracted from sampled records. This involved a five-step process. First, two researchers read each record and independently noted every identified condition, in the original authors’ language. They met weekly to compare their lists for each sampled record. A third team member participated in these discussions to assist in standardizing the coding process and adjudicate any differences in extracted data. This is consistent with how other authors have used a third-member auditing process [25,26]. Discussing disagreements and selection decisions can produce more trustworthy findings [27]. From the original records, conditions were identified from figures or tables or described in text. Included conditions involved any structural, social, individual, or biological determinant that was the result of an empirical study or cited as background, discussion, or in the review of other literature. To begin cleaning this list, step two involved sorting all conditions into rudimentary groups based on similarity (i.e., mental health conditions, social conditions, economic conditions, healthcare conditions, etc.). In step three, we removed duplicate conditions and combined any redundant, synonymous, or similar conditions. This required making subjective decisions about which language to use to name each. In most cases, conditions were named using language from the dataset; in some instances, we used new verbiage that better represented all information that was combined into one condition. The resultant list was reviewed and agreed upon by the three-member content review team. In step four, the names of conditions were printed, cut out, and organized using a pile sorting technique. Pile sorting is a common method used to explore the relationships among contents of a domain [28]. We grouped conditions based on conceptual similarity and inductively named each grouping. This step involved a series of discussions and ongoing edits to determine how conditions and groups were named. Minor editing continued throughout the diagraming process. Finally, step five entailed linking each condition category to one of four social ecological levels. We included the condition description column to be transparent about how we named each condition. To assure relevance, conditions that were only identified in one literary source were not included in the final list.

#### 2.1.4. Diagram Construction

With the final list of conditions and categories, we used the logic of the social ecological model to spatially organize the categories relative to each other. In the first draft of the ecological diagram, arrows were drawn between the categories and checked using a series of logic tests. We assessed the relationship among connected categories by working backwards from the problem (opioid overdose) and asking if downstream conditions logically resulted from upstream conditions. This is similar to the process taught by Renger and Titcomb to determine the sequence and order of the root causes of a health outcome in a visual diagram [17]. Once logical connections between categories were established, we created a full version of the ecological diagram. In the full version, conditions were included within their respective categories. In this version, the arrows reflect the logic of connected categories. No statistical tests were used to assess the relationships among conditions within or across categories. Thus, the diagram does not represent causal relationships as the arrows do not represent empirically confirmed connections.

### 2.2. Subject Matter Expert Diagram Review

#### 2.2.1. Interview Sample

Once completed, we reviewed the full diagram with a group of opioid practitioners who served as third-party subject matter experts (SMEs). SMEs provided a fresh perspective and critiqued the diagram for conceptual coherency and completeness. There is a strong precedent for SME review within qualitative research. For example, SMEs have been used to assess the cultural validity of quality of life measures [29] and the effectiveness of stress-management interventions [30]. They have recommended steps to review military combat deaths [31] and a process for effective safety feedback in a healthcare setting [32]. The latter example was similar to our own, where researchers used literature to establish an initial set of information that was then reviewed with SMEs through interviews [32].

Eleven SMEs were selected from the research teams’ professional network. SMEs were purposively nominated to reflect a diverse range of opioid prevention expertise in academic research, addiction medicine, pharmacy, public health services, health policy, advocacy work, and substance use lived experience. All SMEs were offered $100 for participating and consented to participate in the research. The project was approved by the University of Arizona’s Institutional Review Board.

#### 2.2.2. Interview Data Collection

For the SME interviews, we developed an interview guide and semi-structured process. Before each interview, SMEs were sent a copy of the full diagram and a set of prompts inviting them to reflect on whether they felt conditions were incorrectly included, missing, poorly grouped within categories, or logically incoherent within the diagram. During the interview, SMEs were asked to share thoughts on the content of the diagram (categories, connections, etc.) and the implications of the ecological framework for interventions, including clinical practice and efforts to address the opioid epidemic. Interviews were conducted virtually, using Zoom’s video conferencing platform. This facilitated flexibility in meeting, allowed for screen sharing to visually consult the diagram during the conversation, and the ability to record the audio to generate transcripts from the conversation. Two researchers hosted each interview. One served as the discussion moderator, and the other took detailed notes and asked clarifying follow-up questions. In a semi-structured interview format, the same set of questions was asked to each interviewee, although not necessarily in the same sequence. This allowed for a free-flowing conversation guided by follow-up questions.

#### 2.2.3. Interview Data Analysis

We created interview transcripts by reviewing and correcting the auto-generated Zoom transcripts by listening to the audio recordings. Using the transcriptions, data were extracted using a two-step coding process. First, we coded the transcripts using a set of 18 codes deductively generated from the interview guide. The codes related to how conditions were categorized, how they were connected, recommendations to change listed conditions, diagram formatting, condition changeability, and diagram usability to create interventions that address the opioid crisis. Two team members coded text from each transcript, which they digitally copied into Excel sheets designated for each code. We then reviewed each sheet to identify key themes within each code group. From this, we identified and applied SMEs’ diagram revision recommendations. We also identified SME insights about the challenges and opportunities for using the diagram to create targeted interventions.

## 3. Results

### 3.1. Conditions and Categories

**Figure 2:**
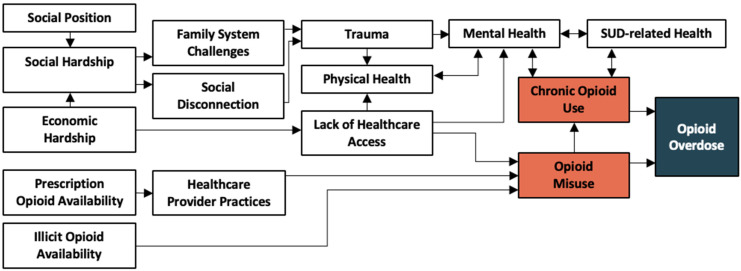
Opioid use, misuse, and overdose condition categories.

In total, we located 226 records. After removing duplicates, 197 were assessed for eligibility and 65 were included. From these, we initially identified 66 unique conditions related to opioid misuse and overdose. These were organized into 16 categories. Figure 2 represents the relationship among these categories. In the full diagram, conditions and categories were included and organized along four social ecological levels. Three categories were located at the social level, four at the community level, three at the relationship level, and five at the individual level. A 16th category included conditions associated with the outcome of opioid overdose. Eleven SMEs were invited to review and comment on the diagram. Nine participated. These included a social worker, a project manager, a data analyst, three researchers, and three clinicians. Four SMEs shared that they had a personal or family history of substance use.

### 3.2. Diagram Revisions

**Figure 3:**
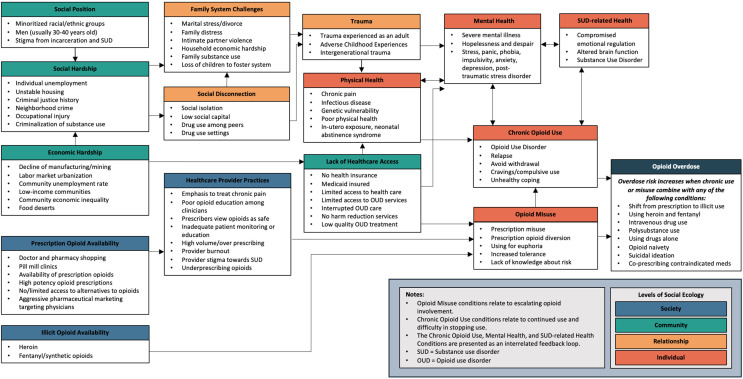
Opioid use, misuse, and overdose conditions categorized by social ecological level.

SME interviews were used to critique and revise the diagram. Suggested changes included adding, combining, and relocating conditions and language and design edits to improve clarity. A total of 40 changes were made to the diagram. Thirteen new conditions were added, and one was split into two, for a total of 80 conditions. Fifteen conditions or categories were renamed, and two were relocated. Finally, six formatting and design changes were made to improve the diagram visually. Figure 3 represents the full diagram and includes all SME-recommended revisions.

From the literature, we identified a few conditions that were not included in the diagram: educational attainment, rurality, gender, and age. It was a challenge to incorporate these in the diagram as numerous sources provided mixed and conflicting evidence. In our sample, opioid overdose was associated with low education attainment [9,10,11,33,34,35,36,37,38,39,40,41] and high educational attainment [12,42,43,44] and with rural residency [10,45,46,47,48] and urban residency [11,46,47,49]. Gender and age were also commonly identified, but they were difficult to separate as they were usually presented in complex and mixed intersections with each other and with race. The one pattern that was consistently described was a relationship between opioid overdose and men around the age of 30–40. We included this condition in the social position category. All diagrammed conditions are listed in Table 1 with source citations and brief descriptions of the information that was used to create and name the condition within the diagram.

### 3.3. Insight on Intervention Strategies Informed by an Ecological Perspective

SMEs were asked to describe conditions they felt were frequently addressed in policy, program, or clinical interventions. Most identified opioid prescribing practices and guidelines. They indicated that opioid prescribing practices have been potentially targeted to the point of overcorrection. Several indicated that it may be counterproductive to continue to crack down on prescription opioids because the opioid crisis has shifted to illicit opioids, like heroin and fentanyl. Because of this, increasing access to naloxone was noted by many to be an important and relatively easy intervention to implement. However, as SMEs noted, an ecological perspective can also highlight different opportunities for intervention, as not all will be easy to implement. As an example, syringe exchanges, like naloxone, also prevent death. Nonetheless, despite demonstrated evidence of reducing bloodborne disease transmission [88], syringe exchanges face political and social hurdles.

SMEs also described conditions they felt were infrequently or under addressed in planned interventions. They pointed to structural factors, such as healthcare access, economic hardship, housing, incarceration, and the illicit drug supply. SMEs shared that these are infrequently delt with because they are considered difficult and expensive to address. They also noted that the US drug policy landscape can exacerbate the opioid crisis. The criminalization of substance use was highlighted as one example. However, when viewing the conditions of opioid use and overdose in an ecological diagram, they noted that it becomes easier to see ways in which systemic issues reinforce and maintain what are commonly thought of as individual-level problems. For example, one SME stated that the effectiveness of treatment and harm reduction measures can be amplified when paired with interventions that target structural factors.

*I think it’s going to be very hard to make any progress [only focusing] downstream…we can do all this work trying to get people mental health care or trying to get them housed…but if that housing [requires] them being abstinent from substances, we are automatically cutting out wide swathes of the population who are either not ready or don’t have a goal of being abstinent from substances. If we’re trying to address some of these things in isolation from actual policy change, I think we’re going to always be fighting an uphill battle*.

—Social worker specializing in SUD/OUD

### 3.4. Insight on Diagram Usefulness and Future Opportunities

Several SMEs indicated that a diagram might be an effective way to communicate information about complex issues, especially issues that involve individuals and groups who have been socially stigmatized or marginalized. A few SMEs noted that diagrams could be used as a communication tool in clinical and public-facing settings. Another offered that the diagram could be useful as an educational or program-planning tool. Because the public and health professionals alike can overly simplify complex issues (often leading to bias and prejudice), an ecological diagram could provide context for the opioid crisis by visually laying out the contributing factors to facilitate a relatable narrative of interconnection.

*If [the diagram] really comes together and simplifies, it could help the general public see these connections, because that’s what you get in your home or on the news or when politicians are talking, you know there’s all kinds of different things that come up. But I don’t think that anyone’s ever seen a document like this that really connects all the dots where they say, ‘Hey…my uncle Jim who died of an opioid overdose and when I look at this document, I can see this is how he started out. He dropped out of college, then he lost his job, then he got divorced, then we found out he had depression,’ you know what I mean? I don’t think that people see all those connections and I think that’s really important for people to understand to really get a better feel for substance use and what it means to have an addiction.*

—OUD intervention program manager at a local health department

SMEs recognized that diagrams require a tradeoff between complexity and usability. For example, they highlighted that the linear set of relationships presented in this diagram may not accurately represent the reality of these conditions and connections, but that it would also be difficult to visually convey their full complexity in a way that would be visually legible. Thus, a balance is required between completeness and coherency. Based on this, SMEs indicated that different or tailored versions of the diagram could be developed to match the needs and capacities of diverse audiences.

## 4. Discussion

From our scoping review and interviews, we identified 80 unique conditions, organized into 16 categories and four social ecological levels. These were identified from 65 literature sources and nine subject-matter-expert interviews. In the interviews, SMEs highlighted opioid prescribing guidelines, naloxone prescribing, and the increase in illicit opioids as well-recognized conditions associated with opioid overdose or overdose prevention. They indicated that underappreciated conditions included healthcare access and economic conditions, like employment and housing. SMEs recognized that individual- and relationship-level conditions tend to be more commonly addressed through program and policy interventions and that community- and social-level conditions tend to be under-addressed. SMEs viewed this as a problem that can be improved by using visual diagrams to illustrate and emphasize often neglected upstream and structural factors.

SMEs indicated that ecological diagrams can provide important context, promote more effective planning and communication, and highlight differences in risks across social groups. But too much information can also be problematic. Complexity needs to be balanced with intuitive and relatable representations of environmental connections. For this, different versions of diagrams can be created to communicate different levels of detail for various audiences. Figure 2 and Figure 3 represent two levels of information complexity. Presenting information at different ecological scales (global, regional, local) and with differing levels of detail are important considerations when considering information management [89]. Future ecological diagrams of opioid misuse might include different organizational schemes that establish connections between the conditions (not just the categories), verify empirical connections between the conditions, or illustrate feedback loops and other non-linear relationships within the health ecology.

To our knowledge, this study is the first effort to scope out and integrate all published descriptions of the social determinants and risk factors of opioid misuse and overdose. However, this is not the first diagramming effort. Other visual models display how individuals enter and exit opioid use disorder [90] and opioid prescribing pathways [91]. Further, a biopsychosocial perspective has been presented on the factors associated with substance consumption and gastrointestinal health [33], as well as a framework of the opioid crisis layered onto the social ecological model [15]. These models highlight important factors of opioid use. We build on these by systematically capturing a broader range of opioid misuse and overdose conditions, sorting them into like groups, and situating them relative to each other with logic-based connections.

In our diagram, the conditions and categories were well distributed across the four social ecological levels. Between three and five categories were represented in each of the four ecological levels. In total, 38 conditions were located in the society and community levels and 34 were located in the relationship and individual levels. This demonstrates a balanced focus in the literature and among SMEs in identifying the antecedent conditions of opioid overdose. Within and outside substance use and opioid literature, social determinants are coming of age [92], and more attention is being given to the causes of the causes [93]. However, SMEs did not view such an equal focus among policy and program efforts to address the opioid crisis. They perceived that most interventions are directed towards addressing more proximal, downstream conditions. This is a long-recognized pattern in public health. Even when downstream conditions are logically and intuitively linked to upstream conditions, the pathways can be long, complex, and multiple and have intervening influences [92]. Identifying upstream determinants is only the first step to change.

However difficult, there is an ethical imperative to address and improve upstream conditions of opioid overdose. Individuals with substance use disorder live socially marginal and isolated [94]. Once a person becomes substance involved, their disadvantage increases if they enter the criminal justice system [95]. The US carceral approach to addressing substance use creates unique risks and prevents the full potential of treatment and harm-reducing assistance to prevent substance-related death and disease [96,97]. As a result, those who use opioids and other illicit substances occupy a stigmatized and constrained social position [98]. An ecological perspective can highlight these systemic factors.

Solutions to the opioid crisis require new, better coordinated, and community-involved ideas [99]. As of 2023, The US Health and Human Service’s overdose prevention strategy emphasizes primary prevention, harm reduction, evidence-based treatment, and recovery support [100]. Efforts in each of these areas would benefit from a more comprehensive understanding of how opioid risk factors are situated relative to each other in a larger health ecology. Examining antecedent conditions vis-à-vis each other makes it easier to examine how factors may co-occur or intersect. This perspective allows health professionals to examine the need for new efforts, more efforts, or a combination of efforts. It also supports questions related to the feasibility of an action, the changeability of conditions, and if change in one area may positively or negatively affect another. For example, when policymakers established restrictive opioid prescribing guidelines, this prevented unnecessary opioid misuse among some individuals and exacerbated harm among others. The restrictions limited options for chronic pain management among older adults [101] and limited access to safer versions of opioids among those who are physiologically dependent. For example, around 2010, in areas with higher prescription rates of Oxycontin reformulations, heroin overdose rates and Hepatitis C infections increased as inveterate users turned to illicit supplies [102].

Using the methods of theory-driven evaluation, health professionals can theorize and check the assumptions that are built into programs, policies, and treatment approaches [103,104]. Making the etiological assumptions explicit makes it easier for planners, implementers, and evaluators to interrogate how they understand the relationship between specific conditions and their occurrence in a larger health ecology. This allows us to question what change is desirable and whether it seems possible [105]. Without considering all potential factors, there is a higher risk that interventions will focus on non-aligned factors or factors that are more obvious but not more influential. Evaluators warn of activity traps, where actions that seem like a good idea for political or other reasons do not address conditions related to the problem [17]. To avoid conceptual cherry-picking, logic-tested diagrams are useful. However, there is an absence of literature describing whether or how visual presentations of complex information have informed policy or funding decisions, including in behavioral health system research [106]. More work is needed in this area.

### Limitations

Our approach was consistent with established criteria for systematic scoping review methodology; nonetheless, we note some potential limitations. We used multiple search strategies to identify and include all available English-language documents, yet it is still possible that documents containing novel determinants were not included in the databases we searched or not published in English. Our objective was to identify and organize all published determinants of opioid use into a coherent ecological diagram. Thus, a systematic assessment of the evidence-base for each identified relationship, while critically important in the future development of this line of inquiry [107], is outside the scope of the current project. Our contribution is a conceptual tool that can guide future inquiries based on a comprehensive ecological diagram of literature-derived determinants and potential relationships among them.

## 5. Conclusions

In this study, we reviewed the literature and interviewed subject matter experts to identify 80 unique conditions of opioid overdose. We organized these into a diagram to offer a visual perspective of opioid overdose and highlight how social, community, relationship, and individual factors may interrelate and contribute to opioid use, misuse, and overdose. Diagrams are useful tools for visually communicating complex sets of information within an ecosystem. Public health practitioners, clinicians, and policy makers may use this diagram as a planning and evaluation tool, to consider more structural or multi-faceted improvement opportunities.

## Figures and Tables

**Table 1:** Opioid use and overdose condition descriptions and categories, by social ecological level.

Society Level	
Categories and Conditions	Condition Descriptions
Illicit Opioid Availability	
Heroin [12,33,37,46,50] Fentanyl/synthetic opioids [41,51,52,53,54,55,56,57,58]	Supply of illicit drugs includes heroin and fentanyl Availability of cheap heroin and fentanyl; cheaper alternatives to prescription opioids, like heroin and fentanyl
Prescription Opioid Availability	
Doctor and pharmacy shopping [36,37,50,59,60] Pill mill clinics [10,34,46,61] Availability of prescription opioids [36,59,62] High-potency opioid prescriptions [36,53,57,58,59,60,62,63,64,65] No/limited access to alternatives to opioids (ALTOs) [50,52,58,64] Aggressive pharmaceutical marketing targeting physicians [10,36,45,46,50,51,52,61,66]	Doctor or pharmacy shopping; number of pharmacies in area Unregulated pain clinics that prescribe many opioids; pill mills; high-volume opioid clinics Availability of longer-acting opioid formulations; hydrocodone and oxycodone Extended-release and long-acting opioids; higher prescribed doses (>100 mg MME) No access to rehabilitation, integrative chronic pain treatment; alternative pain treatments not covered or more expensive than opioids Marketing opioids (such as Oxycontin) for chronic pain; assured safety of high MME opioids
Healthcare Provider Practices	
Emphasis on treating chronic pain [34,36,37,46,52,54,58,61] Poor opioid education among clinicians [36,50,61,67,68] Prescribers view opioids as safe [36,37,45,46,52,61] Inadequate patient monitoring or education [33,39,59] High volume/overprescribing [7,11,33,34,36,37,39,49,50,52,58,59,61,64,65,68,69,70] Provider availability/burnout * Provider stigma towards SUD * Under-prescribing opioids *	Increased attention to pain treatment; pain is 5th vital sign; using opioids as only treatment for pain Ignorance about misuse potential; lack of education among providers, first-responders, staff, and in medical schools View prescription drugs as less dangerous than illicit Insufficient or ineffective oversight of prescription opioids, no discussion of potential adverse effects High-volume opioid prescribing for chronic pain Provider unwilling or unable to treat patients with OUD OUD/SUD viewed as individual failure; restrictive opioid prescribing guidelines may increase stigma Overcorrecting to risk leaves patients with pain less access to medicinal opioids; may turn to illicit sources to manage pain
**Community Level**	
Social Hardship	
Individual unemployment [9,10,35,36,38,40,41,45,46,53,54,56,58,66,71,72,73,74] Unstable housing [9,10,13,35,47] Criminal justice history [9,13,41,47,48,51,58,75] Neighborhood crime [33,35,40] Occupational injury [9,34,51,54,76] Criminalization of substance use *	Unemployment; departure from labor force; low employment success Homelessness; renting; poor housing conditions Overdose highest in incarcerated population; poor access to treatment; low number of addiction specialty courts Neighborhood violence; economic and social factors associated with high incarceration rates Manual labor jobs; risk of disability and chronic pain Criminalization reduces recovery options and resources to improve health outcomes
Social Position	
Minoritized racial/ethnic groups [44,48,51,56,58,74] Men (usually 30-40-years-old) [12,36,38,48,55,56,58,59,74,77] Stigma from incarceration and SUD [51,75]	High prevalence of use and substance use disorder (SUD) among Native Americans and second-generation Hispanics; Black men in their 30s have high death rates and inadequate treatment White men in their 30s have highest incidence of death; White men are two times and black men three times more likely to overdose compared to women Stigma from incarceration and SUD; avoiding treatment
Economic Hardship	
Decline of manufacturing/mining [10,46,54,66] Labor market urbanization [39,45,46,51,59] Community unemployment rate [33,39,49] Low-income communities [33,46,47,56,58] Community economic inequality [9,51,58] Food deserts *	Loss of manufacturing, blue-collar, low-skill, service jobs Jobs shift to urban areas; high-skilled jobs in urban cores; strain on rural labor markets Increased county-level unemployment; decreased labor force participation rate Neighborhood poverty; wage decline; decline in working-class fortunes Economic disadvantage; economic inequality Lack of consistent access to nutritious food
Lack of Healthcare Access	
No health insurance [9,40,51,66] Medicaid insured [33,40,49,51] Limited access to health care [40,45,49,51] Limited access to OUD services [41,47,49,51,57,59,75] Interrupted OUD care [13,40,47,73,75,78] No harm reduction services * Low quality OUD treatment *	Uninsured; loss of insurance; financial barriers to treatment Medicaid; enrollment depends on location Limited primary care services; closing rural hospitals; reliance on emergency departments Opioid agonist treatment prohibited; insufficient access to naloxone, drug prevention programs, or residential treatment Loss of insurance, history of incarceration; release from prison; low adherence in low SES areas; intermittent treatment No syringe-exchange or naloxone-distribution programs; barriers to implementing harm-reduction programs OUD treatment with low evidence-base or poor efficacy
**Relationship Level**	
Family System Challenges	
Marital stress/divorce [9,10,36,38,44,46,53] Family distress [7,10,46,51] Intimate partner violence * Household economic hardship [7,9,10,11,35,38,39,40,41,46,47,51,57,58,59,75] Family substance use * Loss of children to foster system *	Unmarried; divorce Family breakdown; single-parent families due to economic distress Domestic violence; violence exacerbated by SUD/OUD Poverty; limited economic opportunities Family history of substance abuse Loss of children to foster system exacerbates SUD/OUD
Social Disconnection	
Social isolation [7,11,33,42,47,58,66] Low social capital [7,11,40,41,73] Drug use among peers * Drug use settings *	Isolation; loneliness and lack of belonging and sense of purpose; loss of social connection; disconnected from social institutions Poor social support; lack of social capital; limited access to social networks to support treatment Social influence limited to individuals who use drugs Attends social settings where opioids are frequently used
Trauma	
Trauma experienced as an adult [33,66,73,75,79] Adverse childhood experiences [7,33,35,38,41,54,66,67,73,74,80,81] Intergenerational trauma *	Self-medication; psychological trauma Childhood trauma; childhood abuse; high exposure to violence Trauma experienced across generations; systemic racism and discrimination
**Individual Level**	
Physical Health	
Chronic pain [12,33,36,38,42,46,53,56,58,61,63,64,67,73,75,77,78,82] Infectious disease [38,47,75] Genetic vulnerability [33,36] Poor physical health [36,38,47,48,57,58,60,67,73,79,81] In utero exposure *	Self-medication of pain; untreated pain; unclear etiology of pain; learned association with pain relief and opioid use HIV/AIDS infection; immunosuppression; opportunistic infections; poorer physical health Genetics; epigenetics Medical and mental health comorbidities, previous hospitalization, chronic physical conditions Neonatal abstinence syndrome; in utero exposure to opioids potential link with later OUD issues
Mental Health	
Severe mental illness [13,36,50,77] Hopelessness and despair [7,10,11,35,46,51,54,58,61,66,67,71] Stress [33,40,46,61,67,73], panic/social phobia [13,36], impulsivity [33,82], anxiety [33,36,53,54,63,75,77], depression [10,33,36,38,50,53,61,63,64,67,70,73,75,77], post-traumatic stress disorder [63,79,83]	Schizophrenia; bipolar disorder Hopelessness, catastrophic thinking; deaths of despair Anxiety; depression; self-medication of mood; agoraphobia; panic; social phobia; altered neurotransmission in frontostriatal circuit and impulsivity; obsessive drug-seeking; sensitivity to stress; chronic stress; post-traumatic stress disorder
SUD-related Health	
Compromised emotional regulation [33,46,79] Altered brain function [33,58] Substance use disorder [10,37,50,53,65,67,73,74,84]	Anhedonia; compromised emotion regulation; poor emotional well-being; borderline personality disorder Altered neurodevelopment; weakened executive function; alterations of neurocircuitry involving reward History of substance use, misuse, SUD
Chronic Opioid Use	
Opioid use disorder [36,57,60] Relapse [7,9,13,41,57,67,69,75] Avoid withdrawal [67,73] Cravings/compulsive use [66,79] Unhealthy coping [58,61,83]	Opioid addiction; chronic use; use of opioids causing considerable distress Release from prison; history of incarceration; initiating and discontinuing opioid agonist treatment Efforts and desire to avoid or alleviate withdrawal symptoms Compulsive use; craving for prescription drugs Self-medication; chemical coping; no coping skills for pain
Opioid Misuse	
Prescription misuse [37,55,57,67] Prescription opioid diversion [36,37,46,50,52,58,59,61,62,67] Using for euphoria [36,67,73,85] Increased tolerance [50,52,67,75] Lack of knowledge about risk [59,61,67,73,85]	Self-directed prescription increase, dose escalation, and tampering with usage; use without medical supervision Purchasing prescription opioids from dealer; taking opioids prescribed to someone else Reward seeking; desire for euphoria or high; pleasurable initial experience with opioids Increased tolerance; perceived tolerance Perception that opioids are safe; lack of knowledge about use; unaware of differences between opioids; drug substitution
**Opioid Overdose**	
Opioid Overdose (overdose risk increases when chronic use or misuse conditions combine with these conditions)	
Shift from prescription to illicit use [33,37,39,50,52] Using heroin and fentanyl [74,77] Intravenous drug use [13,33,47,57] Polysubstance use [13,47,57,84,86] Using drugs alone [47,75] Opioid naivety [68,73,85] Suicidal ideation [41,58,81] Co-prescribing contraindicated meds [37,47,48,53,57,58,59,60,62,63,64,68,73,77,78,86,87]	Rx functions as gateway drug; transition from Rx opioids to heroin and other illicit opioids Heroin use; synthetic opioids—higher risk of death Taking opioids by injection—overdose risk factor; history of incarceration; sharing needles; using more frequently Any substance abuse; smoking; polysubstance use; alcohol use problem; mixing opioids with other substances Solitary use Drug naivety; opioid naivety Suicidality; suicidal ideation Benzodiazepine, antidepressant, and antipsychotic co-prescribing

* Conditions included based on subject matter expert recommendation.

## Data Availability

No new data were created or analyzed in this study. Data sharing is not applicable to this article.
